# Systematic immunological evaluation in adult patients with inflammatory bowel disease

**DOI:** 10.1186/s12876-026-05072-1

**Published:** 2026-06-29

**Authors:** Ali Can Erdem, Huseyin Ataseven, Mehmet Asıl, Sevket Arslan, Ramazan Ucar, Sadan Soyyigit, Ramazan Dertli

**Affiliations:** 1 Department of Gastroenterology, Faculty of Medicine, Necmettin Erbakan University, Konya, Turkey; 2 Department of Gastroenterology, Anatolia Hospital, Antalya, Turkey; 3 Department of Immunology and Allergy, Faculty of Medicine, Necmettin Erbakan University, Konya, Turkey; 4 Department of Immunology and Allergy, Medova Hospital, Konya, Turkey; 5 Department of Internal Medicine, Faculty of Medicine, Ankara Yıldırım Beyazıt University, Ankara, Turkey

**Keywords:** Inflammatory bowel disease, Immunological evaluation, Primary immunodeficiency, Lymphocyte subsets, Immunoglobulins

## Abstract

**Background:**

Inflammatory bowel disease (IBD) has been frequently reported in patients with primary immunodeficiency; however, the frequency and pattern of immunological abnormalities among adult patients with established IBD remain insufficiently characterized. We aimed to systematically evaluate immunological parameters in adult patients with established IBD. Additionally, we sought to identify clinical factors associated with laboratory-defined immunological deviations.

**Methods:**

In this retrospective observational cohort study, we analyzed 108 adults with Crohn’s disease or ulcerative colitis who underwent structured immunological evaluation as part of routine clinical care between 2011 and 2015 (a period of standardized screening). We excluded patients with major causes of secondary immunodeficiency (e.g., advanced renal failure, HIV infection, severe malnutrition). However, we recorded exposure to medications that could potentially cause secondary immunosuppression. The predefined laboratory panel included serum immunoglobulins, vaccine antibody responses, isohemagglutinin titers (not assessed in blood group AB), peripheral lymphocyte subsets (reported as percentages), and neutrophil phagocytic function, interpreted using standardized reference ranges. Immunological findings were categorized according to the IUIS 2025 phenotypic framework. Multivariable logistic regression was performed to assess factors associated with laboratory-defined immunological deviations.

**Results:**

Clinically significant immunological disorders were identified in 12 patients (11.1%), and laboratory-defined (defined as combined defects across immune compartments) immunological deviations in 60 patients (55.6%). Reduced CD19⁺ B-cell percentages were observed in 45 patients (42.1%) and decreased NK cell percentages in 17 patients (15.9%). In multivariable analysis, increasing age (OR 1.064 per year; 95% CI 1.029–1.100; *p* < 0.001) and male sex (OR 3.61; 95% CI 1.32–9.87; *p* = 0.012) were independently associated with laboratory-defined immunological deviations; medication exposure showed a borderline association (OR 2.78; 95% CI 0.99–7.76; *p* = 0.052).

**Conclusions:**

In this adult IBD cohort evaluated using a structured laboratory framework, measurable immune alterations were common, although most did not meet criteria for clinically significant immunological disorders. These findings highlight immune heterogeneity in adult IBD and support further prospective studies with longitudinal outcomes to clarify the clinical implications and to define which patient subgroups may benefit from targeted immunological assessment.

## Introduction

Inflammatory bowel disease (IBD), encompassing Crohn’s disease and ulcerative colitis, is a chronic immune-mediated disorder characterized by relapsing intestinal inflammation and symptoms such as abdominal pain, diarrhea, bleeding, and malabsorption [1, 2]. The pathogenesis of IBD involves complex interactions among genetic susceptibility, environmental factors, the intestinal microbiota, and dysregulated immune responses within the gastrointestinal tract [3]. Although IBD is traditionally considered a hyperinflammatory condition, several studies have suggested that certain components of the innate and adaptive immune response may also be impaired, raising the possibility that aspects of immune dysfunction contribute to disease susceptibility and progression [4, 5].

Because the gastrointestinal tract represents the largest lymphoid organ in the body, intestinal manifestations are common in patients with primary immunodeficiency (PID) [6]. While traditionally considered pediatric disorders, a substantial proportion of PID cases are diagnosed during adulthood, particularly those involving antibody production defects [7]. This clinical shift is underscored by registry data from the European Society for Immunodeficiencies (ESID), which indicate that the majority of registered PID patients are indeed adults [8]. Gastrointestinal manifestations in PID may include nodular lymphoid hyperplasia, small intestinal bacterial overgrowth, CVID-associated enteropathy, celiac-like disease, and inflammatory bowel disease–like colitis [9]. In this context, “IBD-like” refers to enteropathy that clinically and endoscopically mimics classical IBD but is primarily driven by the underlying inborn error of immunity. Previous studies and a recent systematic review have reported that such IBD-like intestinal inflammation may occur in 3.4% to 61.2% of patients with PID; this wide prevalence range reflects the marked heterogeneity in gastrointestinal involvement across distinct PID subtypes [10].

Despite this recognized association, clinical research has largely approached the relationship from the perspective of identifying IBD-like disease in patients with known immunodeficiency. In contrast, how frequently immunological abnormalities can be detected in adult patients already diagnosed with IBD—remains insufficiently explored [11]. Existing studies have typically focused on selected immunological parameters, specific patient subgroups, or pediatric populations, and systematic immunological evaluation in routine adult IBD cohorts remains limited [11, 12].

Identifying the prevalence and specific patterns of these immunological deviations in patients with established IBD is clinically meaningful. Rather than representing incidental laboratory findings, underlying immune dysregulation may correlate with atypical disease behavior, an increased risk of serious infections, or a treatment-refractory clinical course. Recognizing these alterations early can provide clinicians with vital prognostic clues, helping to identify patient subgroups that may require a risk-stratified approach to immunosuppressive therapies and closer multi-disciplinary surveillance.

In the present study, we systematically evaluated the immunological status of adult patients with IBD using a structured laboratory framework including assessment of humoral immunity, cellular immunity, and phagocytic function. The primary aim of this study was to characterize the spectrum of immunological abnormalities in adult patients with IBD and to identify clinical factors associated with laboratory-defined immunological deviations.

## Materials and methods

### Study design and setting

This retrospective observational cohort study included adult patients with inflammatory bowel disease (IBD) who underwent immunological evaluation as part of routine clinical care at the Immunology and Allergy Diseases Outpatient Clinic between 2011 and 2015. The study protocol was approved by the Ethics Committee for Non-Pharmaceutical and Non-Medical Device Clinical Trials of Necmettin Erbakan University Meram Faculty of Medicine on 04 December 2015 (decision number 2015/376). Following ethical approval, existing clinical and laboratory records were retrospectively reviewed and analyzed. This specific study timeframe corresponds to a period of standardized, structured institutional screening for the underlying postgraduate thesis project, capturing the most complete and uniform immunological dataset available at our center.

No additional interventions or laboratory tests were performed for research purposes.

### Study population

A total of 108 adult patients (≥ 18 years) with a confirmed diagnosis of ulcerative colitis or Crohn’s disease who had undergone immunological evaluation during routine clinical care between 2011 and 2015 were included. All consecutive patients with available immunological laboratory data during the study period were analyzed.

Patients with established major causes of secondary immunodeficiency (including advanced renal failure, nephrotic syndrome, liver failure, HIV infection, severe malnutrition, alcoholism, and protein-losing enteropathy unrelated to IBD) were excluded.

Patients receiving medications that may contribute to secondary immunodeficiency (such as corticosteroids, anti–TNF agents, immunomodulators, or other immunosuppressive therapies) were not excluded but were analyzed separately according to medication exposure (designated as Group II, *n* = 43, versus the unexposed Group I, *n* = 65).

### Immunological assessment

All included patients had undergone a predefined immunological evaluation panel during routine clinical practice, including serum immunoglobulin levels (IgG, IgA, IgM, IgE), vaccine antibody responses (tetanus and pneumococcal antibodies), isohemagglutinin titers (anti-A and/or anti-B; patients with blood group AB were excluded from isohemagglutinin assessment), peripheral lymphocyte subset analysis (CD3⁺, CD4⁺, CD8⁺, CD19⁺, CD16⁺CD56⁺), and neutrophil phagocytic function (phagocytosis assay) (Table 1).

**Table 1:** Reference ranges and classification criteria for immunological parameters

Parameter	Reference Range / Classification	Unit
White blood cell count	4–10	×10³ cells/µL
Neutrophils	1.5–7.3	×10³ cells/µL
Lymphocytes	0.8–5.5	×10³ cells/µL
Immunoglobulin G (IgG)	5–16	g/L
Immunoglobulin M (IgM)	0.4–2.3	g/L
Immunoglobulin A (IgA)	0.7–4.0	g/L
Immunoglobulin E (IgE)	5–150	IU/mL
Tetanus antibody	≥ 0.04	IU/mL
Pneumococcal antibody	≥ 1.0	µg/mL
Isohemagglutinin (anti-A)	≥ 1:16	Titer
Isohemagglutinin (anti-B)	≥ 1:8	Titer
CD16⁺CD56⁺ NK cells (male)	Low < 5%; Normal 5–31.3%; High > 31.3%	% of lymphocytes
CD16⁺CD56⁺ NK cells (female)	Low < 3.5%; Normal 3.5–24.9%; High > 24.9%	% of lymphocytes
CD19⁺ B cells	Low < 6.3%; Normal 6.3–20.8%; High > 20.8%	% of lymphocytes
CD3⁺ T cells (male)	Low < 48%; Normal 48–82.6%; High > 82.6%	% of lymphocytes
CD3⁺ T cells (female)	Low < 56.8%; Normal 56.8–84.1%; High > 84.1%	% of lymphocytes
CD4⁺ T helper cells (male)	Low < 23%; Normal 23–52.6%; High > 52.6%	% of lymphocytes
CD4⁺ T helper cells (female)	Low < 26.9%; Normal 26.9–55.5%; High > 55.5%	% of lymphocytes
CD8⁺ cytotoxic T cells	Low < 12.8%; Normal 12.8–40.2%; High > 40.2%	% of lymphocytes
CD4/CD8 ratio	Low < 0.68; Normal 0.68–3.61; High > 3.61	Ratio

Reference ranges were based on laboratory standards and published adult reference values. Sex-specific cutoff values were applied for lymphocyte subsets where indicated

*NK* indicates natural killer cell, *CD* cluster of differentiation, *Ig* immunoglobulin

Peripheral lymphocyte subsets were reported as percentages of total lymphocytes because absolute subset counts were not consistently available throughout the study period. Sex-specific reference ranges were applied during interpretation to avoid misclassification.

Reference ranges were derived from standardized adult flow cytometry datasets, and percentage-based interpretation is widely used in clinical immunology practice when absolute counts are unavailable. Although absolute counts are the gold standard, percentage-based evaluation provides a robust method for identifying relative shifts in lymphocyte compartments while mitigating the confounding effects of transient fluctuations in total leukocyte counts driven by active inflammation or corticosteroid use. Accordingly, classification in this study was based on established percentage thresholds to ensure internal consistency across the cohort.

Neutrophil function was assessed exclusively by phagocytosis assay and categorized as normal or impaired according to laboratory reference standards. Quantitative oxidative burst and chemotaxis assays were not performed, and we explicitly acknowledge that this represents an incomplete evaluation of the innate phagocytic compartment.

Pneumococcal immunity was evaluated using total (serotype-nonspecific) pneumococcal IgG measurement. Because serotype-specific assays were unavailable, pneumococcal antibody responses were interpreted as screening measures rather than definitive functional assessments.

### Classification of immunological findings

Immunological findings were categorized into two groups: clinically significant immunological disorders and laboratory-defined immunological deviations.

Clinically significant immunological disorders were defined according to the IUIS 2025 phenotypic framework [13], integrating combined abnormalities across humoral, cellular, and innate immune compartments.

Laboratory-defined immunological deviations were operationally defined as cross-sectional, isolated laboratory deviations from established reference ranges that did not meet criteria for a clinically significant disorder, without implying confirmed immunodeficiency or validated prognostic significance. These categories were mutually exclusive; patients fulfilling criteria for a clinically significant immunological disorder were not additionally counted within the laboratory-defined deviations group. The definition was used for descriptive and analytical purposes within the study framework.

### Statistical analysis

All statistical analyses were performed using SPSS (version 22.0; IBM Corp., Armonk, NY, USA). Descriptive statistics were used to summarize demographic and immunological characteristics. Categorical variables were compared using chi-square tests (Monte Carlo simulation when appropriate). Continuous variables were summarized as mean ± standard deviation or median (minimum–maximum), depending on distribution.

To identify independent factors associated with laboratory-defined immunological deviations, multivariable binary logistic regression analysis was performed. The dependent variable was laboratory-defined immunological deviations (yes/no). Independent variables included age, sex, IBD subtype (Crohn’s disease vs. ulcerative colitis), exposure to medications that may cause secondary immunodeficiency (yes/no), and smoking status (current smoker vs. non-smoker). All variables were entered simultaneously (enter method). Odds ratios (OR) with 95% confidence intervals (CI) were reported. Given the modest sample size constraints, the precision of these estimates should be interpreted with caution. A two-sided *p* value < 0.05 was considered statistically significant.

## Results

A total of 108 adult patients with IBD were included in the study. Of these, 81 patients (75.0%) had ulcerative colitis and 27 patients (25.0%) had Crohn’s disease. Overall, 43 patients (39.8%) were receiving medications that may contribute to secondary immunodeficiency (Table 2).

**Table 2:** Immunosuppressive and immunomodulatory medication regimens in Group II (*n* = 43)

Medication Regimen	Number of Patients(*n* = 43)	Percentage (%)
Thiopurine monotherapy (Azathioprine)	25	58.1
Anti-TNF monotherapy (Adalimumab, Infliximab)	6	14.0
Combination therapy (Anti-TNF + Azathioprine)	4	9.3
Other combination therapies (e.g., Azathioprine + Cyclosporine, Anti-TNF + Methotrexate)	3	7.0
Sulfasalazine (Salazopryn) based therapies *	3	7.0
Methotrexate monotherapy	1	2.3
Systemic Corticosteroids (e.g., Methylprednisolone)	1	2.3

*Although sulfasalazine is primarily an anti-inflammatory 5-aminosalicylate (5-ASA) agent, it possesses known immunomodulatory properties and was therefore conservatively grouped within immunomodulatory therapies (Group II) for analytical purposes. Furthermore, monotherapy and combination categories for other agents may include concurrent use of standard 5-ASA derivatives (e.g., mesalazine), which were not independently classified as immunosuppressive

### Humoral immune assessment

Anti-pneumococcal IgG antibody levels were measured in all patients and were ≥ 1.0 µg/mL in all cases, indicating an adequate humoral response. Tetanus antibody responses were sufficient in the majority of individuals, with 83.3% (*n* = 90) demonstrating protective antibody levels. However, 18 patients had no evaluable vaccine response because they had not received tetanus vaccination or a booster dose, precluding evaluation of vaccine response.

Isohemagglutinin testing was performed in 96 patients after exclusion of individuals with blood group AB. Adequate isohemagglutinin responses were observed in 88 patients (91.7%). Serum immunoglobulin analysis revealed no cases of IgG deficiency. Reduced IgM levels were observed in 8 patients (7.4%), reduced IgA levels in 4 patients (3.7%), and elevated IgE levels in 6 patients (5.6%). Overall, abnormalities in at least one immunoglobulin parameter were detected in 12 patients (11.1%) (Table 3).

**Table 3:** Serum immunoglobulin levels in patients with inflammatory bowel disease

Immunoglobulin	Category	Group I (*n* = 65) (%)	Group II (*n* = 43) (%)	Total (*n* = 108) (%)
IgG	Normal	56 (86.2)	34 (79.1)	90 (83.3)
	High	9 (13.8)	9 (20.9)	18 (16.7)
IgM	Low	6 (9.2)	2 (4.7)	8 (7.4)
	Normal	57 (87.7)	38 (88.4)	95 (88.0)
	High	2 (3.1)	3 (7.0)	5 (4.6)
IgA	Low	2 (3.1)	2 (4.7)	4 (3.7)
	Normal	63 (96.9)	41 (95.3)	104 (96.3)
IgE*	High	4 / 45 (8.9)	2 / 31 (6.5)	6 / 76 (7.9)
	Normal	41 / 45 (91.1)	29 / 31 (93.5)	70 / 76 (92.1)
Overall serum Ig assessment	Normal	57 (87.7)	39 (90.7)	96 (88.9)
	Any abnormality	8 (12.3)	4 (9.3)	12 (11.1)

All patients underwent serum IgG, IgM, and IgA testing. Serum IgE levels were measured in a subset of patients (Group I, n=45; Group II, n=31; total n=76). Percentages for IgE are calculated using tested denominators. Reference ranges were IgG 5–16 g/L, IgM 0.4–2.3 g/L, IgA 0.7–4.0 g/L, and IgE 0–150 IU/mL. Group I includes patients not using medications that may cause secondary immunodeficiency, and Group II includes patients using such medications. *Ig* indicates immunoglobulin

### Cellular immune assessment

Peripheral lymphocyte subset analysis demonstrated abnormalities across several immune compartments (Table 4). Reduced CD19⁺ B-cell percentages were observed in 45 patients (42.1%), while decreased NK cell percentages (CD16⁺CD56⁺) were detected in 17 patients (15.9%). T-cell subsets (CD3⁺, CD4⁺, and CD8⁺) were largely within reference ranges, with only a small proportion of patients demonstrating reduced CD3⁺ or CD4⁺ T-cell percentages. Overall, half of the cohort exhibited at least one abnormality in peripheral lymphocyte subsets.

**Table 4:** Peripheral lymphocyte subset values in patients with inflammatory bowel disease

Peripheral Lymphocyte Subset	Category	Group I (*n* = 65) n (%)	Group II (*n* = 43) n (%)	Total (*n* = 108) n (%)
CD16⁺CD56⁺ NK cells	Normal	57 (87.7)	33 (78.6)	90 (84.1)
	Low	8 (12.3)	9 (21.4)	17 (15.9)
CD19⁺ B cells	Normal	42 (64.6)	17 (40.5)	59 (55.1)
	Low	22 (33.8)	23 (54.8)	45 (42.1)
	High	1 (1.5)	2 (4.8)	3 (2.8)
CD3⁺ T cells	Normal	62 (95.4)	41 (97.6)	103 (95.4)
	Low	3 (4.6)	1 (2.4)	4 (3.7)
CD4⁺ T cells	Normal	63 (96.9)	41 (97.6)	104 (97.2)
	Low	2 (3.1)	1 (2.4)	3 (2.8)
CD8⁺ T cells	Normal	54 (83.1)	35 (83.3)	89 (83.2)
	High	11 (16.9)	7 (16.7)	18 (16.8)
CD4/CD8 ratio	Normal	56 (86.2)	39 (92.9)	95 (88.8)
	Low	7 (10.8)	2 (4.8)	9 (8.4)
	High	2 (3.1)	1 (2.4)	3 (2.8)
Neutrophil phagocytic function	Normal	65 (100)	42 (97.7)	107 (99.1)
	Low	0	1 (2.3)	1 (0.9)
Overall PLS assessment	Normal	38 (58.5)	16 (37.2)	54 (50.0)
	Abnormal	27 (41.5)	27 (62.8)	54 (50.0)

Peripheral lymphocyte subsets were classified as low, normal, or high according to sex-specific percentage-based reference ranges. Absolute lymphocyte subset counts were not consistently available during the study period; therefore, percentage-based interpretation was applied uniformly across the cohort. Sex-specific cutoff values were applied during interpretation but are not shown separately in the table for clarity. Neutrophil function was assessed by phagocytosis assay only; results are reported as normal or impaired. Cutoffs are provided in Table 1. Group I includes patients not using medications that may cause secondary immunodeficiency, and Group II includes patients using such medications

*NK* indicates natural killer cell, *CD* cluster of differentiation, *PLS* peripheral lymphocyte subsets

Neutrophil phagocytic function was assessed in all patients, and impaired phagocytic activity was identified in one patient (0.9%); however, this finding remains constrained by the limited scope of the assay.

### Immunological classification

**Fig. 1:**
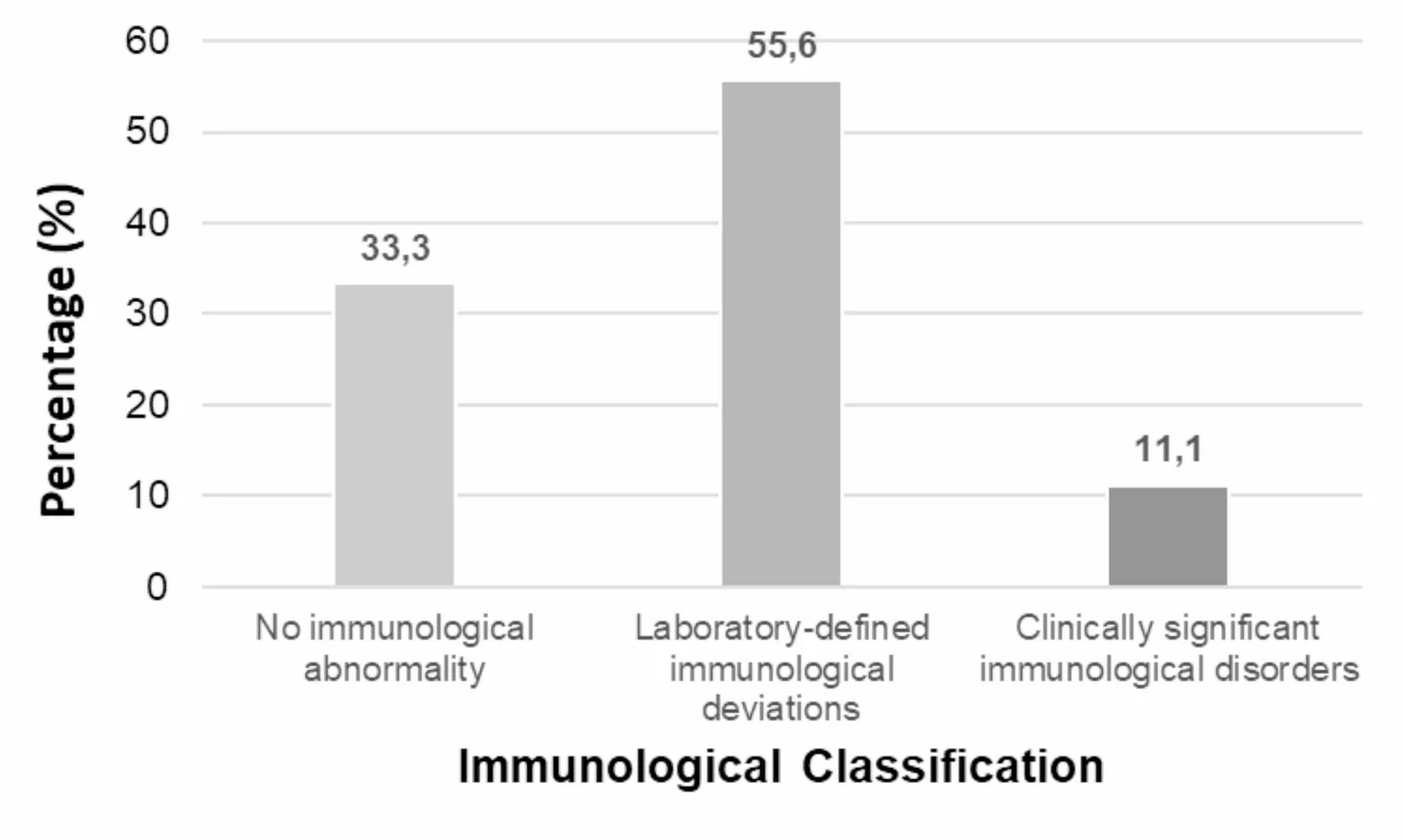
Overall distribution of immunological findings in adult patients with IBD. Patients were classified into three categories according to the IUIS 2025 framework: no immunological abnormality, laboratory-defined immunological deviations, and clinically significant immunological disorders

When humoral, cellular, and innate immune findings were evaluated together, clinically significant immunological disorders were identified in 12 patients (11.1%), primarily reflecting combined B-cell abnormalities associated with immunoglobulin deficiencies and/or functional antibody defects. Detailed patterns of combined immunological abnormalities are presented in Table 5. According to the overall classification based on the IUIS 2025 framework, 36 patients (33.3%) had no immunological abnormality, 12 patients (11.1%) had clinically significant immunological disorders, and 60 patients (55.6%) demonstrated laboratory-defined immunological deviations (Table 6; Fig. 1).

**Table 5:** Distribution of immunological abnormality patterns in patients with inflammatory bowel disease

Category of Immunological Pattern	Specific Abnormality	Group I *n* = 65 (%)	Group II *n* = 43 (%)	Total *n* = 108 (%)
Normal findings	Normal	25 (38.5)	11 (25.6)	36 (33.3)
Isolated quantitative abnormalities	Low CD19	8 (12.3)	7 (16.3)	15 (13.9)
	IgM deficiency	1 (1.5)	0	1 (0.9)
	IgA deficiency	1 (1.5)	0	1 (0.9)
	IgE elevation	1 (1.5)	0	1 (0.9)
	Low CD3	1 (1.5)	0	1 (0.9)
Isolated functional abnormalities	Low isohemagglutinin response	9 (13.8)	6 (14.0)	15 (13.9)
	Low NK cell percentage (CD16⁺CD56⁺)	0	4 (9.3)	4 (3.7)
Combined immunological abnormalities	Low CD19 + IgA deficiency	1 (1.5)	1 (2.3)	2 (1.9)
	Low CD19 + IgM deficiency	0	1 (2.3)	1 (0.9)
	Low CD19 + Low NK cell percentage	1 (1.5)	3 (7.0)	4 (3.7)
	Low CD19 + Low CD4/CD8 ratio	2 (3.1)	2 (4.7)	4 (3.7)
	Low CD19 + Low isohemagglutinin response	1 (1.5)	1 (2.3)	2 (1.9)
	Low CD19 + Low NK cell percentage + IgM deficiency	1 (1.5)	1 (2.3)	2 (1.9)
	Low CD19 + Low isohemagglutinin response + IgM deficiency	2 (3.1)	0	2 (1.9)
	Low CD19 + Low isohemagglutinin response + IgE elevation	1 (1.5)	1 (2.3)	2 (1.9)
	Low NK cell percentage + Low isohemagglutinin response	0	2 (4.7)	2 (1.9)
	Low CD4 + Low CD4/CD8 ratio + Low isohemagglutinin response	1 (1.5)	0	1 (0.9)
	Low CD3 + IgE elevation	0	1 (2.3)	1 (0.9)
	Low CD19 + Low CD4 + Low isohemagglutinin response	0	1 (2.3)	1 (0.9)

Immunological patterns were defined based on combined abnormalities across humoral, cellular, and innate immune compartments rather than isolated laboratory findings. Frequencies reflect cross-sectional laboratory patterns and do not imply longitudinal clinical progression. Group I includes patients not using medications that may cause secondary immunodeficiency, and Group II includes patients using such medications

*Ig* indicates immunoglobulin, *CD* cluster of differentiation, *NK* natural killer cell

**Table 6:** Overall immunological classification of the study cohort according to the IUIS 2025 framework

Category	Total (*n* = 108) (%)	Group I (*n* = 65) (%)	Group II (*n* = 43) (%)
No immunological abnormality	36 (33.3)	25 (38.5)	11 (25.6)
Clinically significant immunological disorder	12 (11.1)	8 (12.3)	4 (9.3)
Laboratory-defined immunological deviations	60 (55.6)	32 (49.2)	28 (65.1)

Overall classification was based on the IUIS 2025 classification, integrating combined immunological abnormalities, functional immune defects, and relevant clinical features, and reflects a cross-sectional assessment. Group I includes patients not using medications that may cause secondary immunodeficiency, and Group II includes patients using such medications

Among patients with clinically significant immunological disorders, 8 patients (12.3%) were in Group I and 4 patients (9.3%) were in Group II. In contrast, laboratory-defined immunological deviations were observed in 32 patients (49.2%) in Group I and 28 patients (65.1%) in Group II (Table 6).

### Factors associated with laboratory-defined immunological deviations

Multivariable logistic regression analysis was performed to evaluate factors associated with laboratory-defined immunological deviations. Increasing age was independently associated with higher odds of laboratory deviations (OR 1.064 per year; 95% CI 1.029–1.100; *p* < 0.001). Male sex was also independently associated with the outcome (OR 3.61; 95% CI 1.32–9.87; *p* = 0.012). Use of medications that may cause secondary immunodeficiency showed a borderline association with the overall composite outcome of laboratory deviations (OR 2.78; 95% CI 0.99–7.76; *p* = 0.052), whereas IBD subtype and smoking status were not independently associated with laboratory deviations (Table 7).

**Table 7:** Multivariable logistic regression analysis of factors associated with laboratory-defined immunological deviations

Variable	Odds Ratio (OR)	95% Confidence Interval	*p* value
Age (per 1-year increase)	1.064	1.029–1.100	< 0.001
Male sex (vs. female)	3.61	1.32–9.87	0.012
Crohn disease (vs. ulcerative colitis)	0.97	0.31–3.01	0.961
Use of medications that may cause secondary immunodeficiency (yes vs. no)	2.78	0.99–7.76	0.052
Smoking (current smoker vs. non-smoker)	0.59	0.22–1.62	0.308

Odds ratios were derived from a multivariable binary logistic regression model for laboratory-defined immunological deviations (yes/no), as defined in Table 6, with all variables entered simultaneously. *OR* odds ratio, *CI* confidence interval

Comparison of laboratory parameters according to medication exposure demonstrated largely similar immunological profiles between groups. Among the evaluated parameters, only CD3⁺ T-cell levels differed significantly between patients receiving and not receiving medications associated with secondary immunodeficiency (*p* = 0.005).

These findings indicate that measurable immune alterations were common in this adult IBD cohort despite the absence of overt clinical suspicion of immunodeficiency.

## Discussion

Intestinal involvement is a well-recognized feature of primary immunodeficiency disorders and is routinely addressed during PID follow-up [14]. In contrast, IBD is usually managed from a gastroenterology-centered perspective, and structured immunological evaluation is not part of routine IBD care [15].

The present study addresses a clinically underexplored question: how frequently measurable immune alterations (encompassing deviations across humoral, cellular, and innate compartments) can be detected in adult patients with IBD when systematically evaluated outside the classical suspicion-driven paradigm. Rather than examining IBD occurrence in known immunodeficiency, we retrospectively evaluated a structured laboratory framework in a routine IBD cohort. Using a predefined laboratory-based framework encompassing humoral immunity, cellular immunity, and phagocyte function—after excluding major causes of secondary immunodeficiency—we identified clinically significant immunological disorders (11.1% of the cohort) as well as laboratory-defined immunological deviations (55.6%), despite the absence of overt clinical suspicion of immunodeficiency within this routine clinical practice cohort (Table 6; Fig. 1). Furthermore, the presence of these systemic immune alterations may also reflect the complex interplay between local intestinal immunity and the gut microbiome. Dysbiosis in the gut microbiota is known to profoundly influence intestinal immune homeostasis, potentially driving both local and systemic immune dysregulation. Consequently, the measurable immune deviations observed in our cohort might represent a broader breakdown in mucosal tolerance and host-microbiome cross-talk, which may be associated with perpetuated chronic inflammatory activity and influence disease progression [16].

Given the observational design and the focus on cross-sectional immunological patterns, extensive sex-stratified analyses were not performed due to sample size constraints, beyond inclusion of sex as a covariate. Most immunological parameters did not differ between patients receiving and not receiving medications associated with secondary immunodeficiency, suggesting that immune alterations observed in this cohort cannot be fully explained by treatment exposure alone.

From a practical gastroenterology perspective, our findings support a risk-stratified approach to immunological testing in adult patients with IBD rather than indiscriminate screening [17]. Initial clinical assessment may include careful review of infection history, treatment response, and basic laboratory parameters such as complete blood count and serum IgG, IgA, and IgM levels. In selected patients with suggestive clinical or laboratory features, more detailed immunological assessment in collaboration with immunology specialists may be considered [18]. However, the present study does not establish the clinical utility or outcome impact of routine immunological screening in all patients with IBD. These findings should be interpreted as hypothesis-generating and do not support indiscriminate immunological screening in all adult patients with IBD.

Our multivariable analysis identified independent factors associated with laboratory-defined immunological deviations. Increasing age and male sex remained significantly associated with laboratory deviations after adjustment for IBD subtype, smoking, and medication exposure (Table 7). These findings are cross-sectional and do not imply causality; furthermore, adjustment was limited to a prespecified set of variables. The borderline association observed for medication exposure suggests that treatment-related immune modulation may contribute in some patients; however, the absence of strong independent associations for IBD subtype and smoking indicates that immune deviations in adult patients with IBD likely reflect heterogeneous and multifactorial mechanisms [19, 20].

The isolated difference in CD3⁺ T-cell levels observed between patients receiving and not receiving immunosuppressive therapy should be interpreted with caution. This finding may reflect treatment-related T-cell modulation or redistribution of lymphocytes from peripheral blood to inflamed intestinal tissue or other IBD-specific factors, rather than systemic organ failure or a primary T-cell defect [21]. Importantly, this difference was not accompanied by parallel abnormalities in other T-cell subsets or by clinical features suggestive of combined immunodeficiency [22].

With current treatments, the life expectancy of patients with IBD is increasing, and disease duration has consequently extended. As age and disease duration increase, factors such as IBD-related protein-losing enteropathy or similar disease-specific mechanisms may contribute to reduced immunoglobulin levels [23, 24] Age-related immunological changes may also influence IgA and IgM concentrations [23].

In our cohort, a subset of adult patients with IBD exhibited selective immunoglobulin abnormalities, including IgM deficiency (7.4%), IgA deficiency (3.7%), and elevated IgE levels (5.6%), although none demonstrated IgG deficiency or fulfilled diagnostic criteria for common variable immunodeficiency (CVID) (Table 3). Importantly, these selective abnormalities were largely isolated findings without accompanying cellular or innate immune defects. From the perspective of the IUIS 2025 classification, the absence of hypogammaglobulinemia does not exclude phenotypically defined immune alterations [13]. In this study, classification was based on combined laboratory findings across immune compartments. However, these classifications reflect cross-sectional laboratory patterns and should not be interpreted as evidence of progressive or clinically confirmed inborn errors of immunity.

Reduced CD16⁺CD56⁺ and CD19⁺ cell percentages were observed in a subset of patients, including some with clinically significant immunological disorders (Table 5). However, the majority of patients with these changes did not meet criteria for clinically significant disorders, reinforcing the distinction between laboratory and clinical significance.

The absence of parallel immunoglobulin deficiencies in many patients with reduced CD19 expression may reflect preserved immunoglobulin production in lymphoid tissues outside the peripheral blood [25, 26].

Previous reports have indicated that IBD may occur in 19% to 32% of patients with selected primary immunodeficiency disorders [26, 27]. When viewed from the opposite perspective, our findings suggest that immune alterations may also be detectable in a subset of adult patients with IBD. This study highlights the bidirectional relationship between IBD and immune system abnormalities and underscores the importance of clinical awareness, particularly in patients with atypical disease course or recurrent infections. Nevertheless, data describing systematic immune system evaluation in adult IBD cohorts remain limited, particularly in routine clinical settings. These observations may have practical implications for gastroenterology practice, as recognition of underlying immune abnormalities could contribute to earlier identification of patients with atypical disease behavior, recurrent infections, or treatment-refractory clinical courses.

In addition to clinically significant immunological disorders, a substantial proportion of patients demonstrated laboratory-defined immunological deviations that did not fulfill criteria for overt immunodeficiency. These deviations were defined operationally based on established laboratory reference ranges and were used for descriptive and analytical purposes within this study framework. Because longitudinal outcome data were not available, the clinical trajectory and long-term clinical significance of these laboratory findings remain uncertain.

Several limitations should be acknowledged. First, the retrospective single-center design and modest sample size limit generalizability. Accordingly, the present findings should be interpreted primarily as hypothesis-generating observations that may inform the design of future prospective studies evaluating immune system alterations in adult IBD populations. Second, no healthy control group was included; therefore, comparisons relied on established laboratory reference ranges. Because the primary aim of the present study was to characterize laboratory-based immunological patterns within an adult IBD cohort rather than to perform direct case–control comparisons, interpretation relied on established clinical reference ranges routinely used in immunological practice. Third, longitudinal clinical outcomes were not assessed, precluding evaluation of whether identified laboratory deviations were associated with subsequent infections, treatment modifications, or progression to overt immunodeficiency. In addition, peripheral lymphocyte subsets were analyzed using percentage-based thresholds rather than absolute counts, which may limit direct comparison with studies reporting absolute cell numbers.

Finally, systematic genetic testing was not performed, and immunological classification relied on phenotypic and functional laboratory criteria in accordance with the IUIS 2025 framework [13]. Future prospective, multicenter studies incorporating standardized immune assays and longitudinal outcome assessment are required to clarify the clinical relevance of these findings.

In conclusion, structured laboratory evaluation revealed a substantial frequency of measurable immune alterations in adult patients with IBD. Although most abnormalities did not meet criteria for overt immunodeficiency, these findings highlight the heterogeneity of immune profiles in adult IBD and emphasize the importance of clinical awareness when evaluating patients with atypical disease course or recurrent infections. Future prospective studies incorporating longitudinal follow-up are needed to further clarify the clinical implications of these immune alterations.

## Data Availability

The datasets used and/or analysed during the current study are available from the corresponding author on reasonable request.

## Abbreviations

IBD: Inflammatory bowel disease
PID: Primary immunodeficiency
NK: Natural killer
CVID: Common variable immunodeficiency
